# Homeodomain-only protein suppresses proliferation and contributes to differentiation- and age-related reduced CD8^+^ T cell expansion

**DOI:** 10.3389/fimmu.2024.1360229

**Published:** 2024-02-12

**Authors:** Qian Yang, Michael Patrick, Jian Lu, Joseph Chen, Yongqing Zhang, Humza Hemani, Elin Lehrmann, Supriyo De, Nan-ping Weng

**Affiliations:** ^1^ Laboratory of Molecular Biology and Immunology, National Institute on Aging, National Institutes of Health, Baltimore, MD, United States; ^2^ Laboratory of Genetics and Genomics, National Institute on Aging, National Institutes of Health, Baltimore, MD, United States

**Keywords:** T cell activation, T cell proliferation, negative regulator, T cell differentiation, aging

## Abstract

T cell activation is a tightly controlled process involving both positive and negative regulators. The precise mechanisms governing the negative regulators in T cell proliferation remain incompletely understood. Here, we report that homeodomain-only protein (HOPX), a homeodomain-containing protein, and its most abundant isoform *HOPXb*, negatively regulate activation-induced proliferation of human T cells. We found that *HOPX* expression progressively increased from naïve (T_N_) to central memory (T_CM_) to effector memory (T_EM_) cells, with a notable upregulation following *in vitro* stimulation. Overexpression of *HOPXb* leads to a reduction in T_N_ cell proliferation while *HOPX* knockdown promotes proliferation of T_N_ and T_EM_ cells. Furthermore, we demonstrated that HOPX binds to promoters and exerts repressive effects on the expression of *MYC* and *NR4A1*, two positive regulators known to promote T cell proliferation. Importantly, our findings suggest aging is associated with increased *HOPX* expression, and that knockdown of *HOPX* enhances the proliferation of CD8^+^ T cells in older adults. Our findings provide compelling evidence that HOPX serves as a negative regulator of T cell activation and plays a pivotal role in T cell differentiation and in age-related-reduction in T cell proliferation.

## Introduction

T cell activation and proliferation are intricately regulated processes involving a balance of positive and negative regulators. This balance is crucial to ensure effective immune protection against pathogens while minimizing the risk of self-injury (1, 2). The interaction of T cells and antigen presenting cells, mediated by the T cell receptor (TCR)/antigen/MHC complex, coupled with engagement of co-stimulatory receptors, leads to T cell activation. Positive transcriptional factors, such as MYC, are activated to promote T cell proliferation. Conversely, TCR engagement in the absence of co-stimulatory receptor engagement can result in T cell anergy or inactivation (3–5). Furthermore, the engagement of co-inhibitory receptors triggers the activation of molecules like SHPs and PP2A and inhibits the activities of AKT, PLCγ, and PKCθ which are essential for T cell activation and proliferation (6). However, it remains less clear whether engagement of TCR and co-stimulatory receptor activates negative regulators in this context.

The homeodomain-only protein (HOPX) is a transcriptional cofactor known for its role in regulating cell fate decisions (7, 8). It also functions as a tumor suppressor gene in multiple forms of cancer (9, 10). *HOPX* exists in several alternative splicing variants (11), and does not directly bind to DNA. Instead, it forms complexes with co-factors, such as serum response factor (SRF), to modulate gene expression (8, 12). Recent studies have unveiled additional roles of Hopx in immune function. It has been shown to be essential for the survival of Th1 effector/memory cells (13) and is induced following interaction with dendritic cells (DCs) in regulatory T cells (Tregs). This induction promotes DC-mediated T cell unresponsiveness (14). Furthermore, *HOPX* has been found to modulate Treg responsiveness in autoimmune animal models (15). During COVID-19 infection, there is notable clonal expansion of CD8^+^ T cells in the lungs of patients and these expanded T cells exhibit high expression of *HOPX* (16). Despite these findings, the function of HOPX in T cells, particularly in humans, has not been comprehensively investigated.

In this study, we have demonstrated that *HOPX* expression exhibits an increase in human T cells transitioning from naïve (T_N_) to memory T cells, encompassing both central memory (T_CM_) and effector memory (T_EM_) cells. Furthermore, we observed its induction following *in vitro* activation via anti-CD3/CD28 antibodies. In human CD8^+^ T cells, we identified the presence of three *HOPX* isoforms, with *HOPXb* being the dominant isoform. Functional experiments revealed that overexpression of *HOPXb* partially impedes T cell proliferation, while the knockdown of HOPX leads to enhanced T cell proliferation. Mechanistically, HOPX achieves this repression of activation-induced proliferation by inhibiting the expression of key regulators, namely MYC and NR4A1. Lastly, we found that *HOPX* expression levels were 1.5-fold higher in older subjects, suggesting its role as a regulator in aging T cells.

## Materials and methods

### PBMCs isolation, T cell subsets enrichment, and cell culture

Seventy-four healthy blood donors were recruited under NIH IRB approved protocols and all donors provided written informed consent regarding their participation in the study. Peripheral Blood Mononuclear Cells (PBMCs) were isolated from blood by Ficoll-Hypaque density gradient centrifugation (**Supplementary Table 1**). Cells were stained for sorting with anti-CD8, anti-CD4, anti-CD62L, and anti-CD45RA. MoFlo XDP Sorter (Beckman Coulter) was used to sort CD45RA^+^ CD62L^+^ naïve cells (T_N_), CD45RA^-^ CD62L^+^ central memory (T_CM_) cells, and CD45RA^-^ CD62L^-^ effector memory (T_EM_) cells. All cells were maintained at 37 °C and 5% CO_2_. The human leukemic T-cell line Jurkat, subclone E6 (ATCC), was maintained in standard growth medium (RPMI 1640 medium supplemented with 10% fetal bovine serum) while 293T cell line (ATCC) was cultured in DMEM/HG media with 10% FBS. Passages 5-15 were used in this study.

### Over-expression and knock-down of HOPX in Jurkat and primary T cells by lentiviral transduction

The *HOPX (a,b,c)* sequences were synthesized by Genewiz and then converted into lentiviral transfer vector pLVX-IRES-tdTomato (Clontech) under the CMV promoter using EcoRI (5′) and BamHI (3′). We used an empty transfer vector pLVX-IRES-tdTomato as control. The overexpression efficiency of each construct was tested *in vitro* with fluorescence and verified by qPCR. The shRNA sequence targeted on *HOPX* (GACCCAGAAATGGTTTAAGCA) was synthesized by Millipore Sigma and then cloned into the vector pLKO.3G_X7 under U6 promoter using AgeI (5’) and EcoRI (3’). The vector pLKO.3G_X7 (Addgene) was used as shRNA control, and contained an EGFP reporter. The knock-down efficiency of each construct was tested *in vitro* with EGFP fluorescence and verified by Western blot. To produce lentiviruses, 293T cells were initially plated into a p10 dish and then transfected at around 80% confluence with 1 µg of VSVG (envelope vector), 8 µg of dR8.2 (packaging vector), and 10 µg of the transfer vector in the presence of 50 μL of FuGENE (Promega). The viral supernatant was collected after 48 hours, cleared by centrifugation with 1500 rpm for 5 min at 4°C, and then passed through a 0.45 μm pore PVDF Millex-HV filter (Millipore). Lentivirus was concentrated using PEG solution (5X) and centrifuged at 2800 rpm for 1 hour. The virus pellets were resuspended in 100 μL PBS and stored at -80°C until use. CD4^+^ T cells and CD8^+^ T cells were stimulated with anti-CD3/CD28 conjugated microbubbles one day before transduction. Lentiviral stocks were added to either primary T lymphocytes or Jurkat T cells cultured in RPMI 1640 media supplemented with 10% FBS and 5 μg/mL polybrene. After overnight incubation, lentivirus-containing supernatant was removed by centrifugation and fresh media was added. Transduced T cells were harvested for examination of the expression of tdTomato by flow cytometry (BD FACSCantoII) at the indicated time points. tdTomato^+^CD8^+^ T cells (both *HOPX* and control virus) were sorted by BD FACSAria Fusion on Day 3 and used for gene expression analysis by gene expression microarrays (Agilent).

### Construction and stimulation of TCR reporter cell line

The entire V regions of TCR a and b (TRAV8-6-TRAJ42) chains were synthesized (Genewiz) and then cloned into the transfer vector pHAGE (Addgene) containing the mCherry reporter. The lentivirus was produced using the same method described above. Then TCR was transduced into the NJ76 cell line. The stable NJ76-TRAV8-6-TRAJ42 cell line was sorted based on mCherry. After overexpression of HOPX or control, TCR was stimulated with anti-CD3/CD28 for 4 hours and 24 hours. The GFP signal was detected with flow cytometry and analyzed with FlowJo.

### Gene expression analysis by real-time RT-PCR

Total RNA was isolated with RNeasy Mini Kit (QIAGEN) according to the manufacturer’s instructions. One microgram of total RNA was reverse transcribed to cDNA using the SuperScript™ IV First-Strand Synthesis System kit (Invitrogen). cDNA was subsequently subjected to SYBR Green-based real-time PCR using an ABI 7900 Real-time PCR System (Applied Biosystems). Primers used in real-time PCR are shown in **Supplementary Table 2**. All results were obtained from at least three independent experiments. *ACOX1* was used as an internal control.

### Microarray analysis of gene expression

Total RNA was extracted from tdTomato^+^CD8^+^ T cells (n=4 donors) sorted by BD FACSAria Fusion on Day 3 using the RNeasy Mini Kit (QIAGEN) according to the manufacturer’s instructions and stored at -80°C. RNA integrity and concentration were evaluated with the Bioanalyzer 2100 and RNA 6000 Nano chip (Agilent Technologies). Cyanine-3 (Cy3) labeled cRNA was prepared from 200 ng total RNA using the Low-Input Quick Amp Labeling Kit (Agilent, Santa Clara, CA) according to the manufacturer’s instructions, followed by RNeasy column purification (QIAGEN, Valencia, CA). Dye incorporation and cRNA yield were evaluated with the NanoDrop ND-1000 Spectrophotometer (Wilmington, DE). A total of 300 ng Cy3-labeled cRNA and 300 ng of a Cy5-labeled universal standard was fragmented at 60°C for 30 minutes in a reaction volume of 25 μL containing 0.5x Agilent fragmentation buffer and 2x Agilent gene expression blocking agent. After fragmentation, 25 μL of 2× Hi-RPM Hybridization Buffer was added to the fragmentation mixture and hybridized to Agilent SurePrint G3 Human GE v3 8x60K Microarray gene expression microarrays for 17 hours at 65°C in a rotating Agilent hybridization oven. After hybridization, microarrays were rinsed one minute at room temperature in GE Wash Buffer 1 (Agilent) and one minute in 37°C GE Wash buffer 2 (Agilent) and dried by slowly removing from Wash buffer 2. Following post-hybridization rinses, arrays were scanned using a dual-intensity Agilent SureScan microarray Scanner at 5 micron, and hybridization intensity data extracted from the scanned images using Agilent’s Feature Extraction Software 9.1 (Agilent) using default parameters to obtain background subtracted and spatially detrended Processed Signal intensities.

Extraction software with default settings was used to obtain normalized expression values from the raw scans. Normalization modified the raw intensity values to compensate for the different dye efficiency in two-channel microarray experiments using Cy3-labeled samples and universal Cy5-standard. A typical volcano plot shows the Z transformed data (Z ratio) on the x-axis and minus log10 of the p-value on the y-axis. Significant differentially expressed genes were defined as Z ratio > 1.5 or < -1.5 and p < 0.01. Functional profiling was performed as Reactome pathway analysis (17, 18). We applied Normalized Enrichment Score (NES) > 1.5 or < -1.5 and p < 0.01 to identify significantly changed pathways. Raw and normalized data are deposited to the Gene Expression Omnibus (GEO) repository accession number GSE234697.

### ChIP-qPCR of HOPX binding

Approximately 10^7^ of fresh and activated CD8^+^ T cells were pooled. Cells were crosslinked in 1% formaldehyde at room temperature for 10 minutes. After quenching crosslinking for 5 minutes with 125 mM glycine and washing the cells in cold PBS, cells were lysed in 50 mM Tris-Hcl, pH 8.0, 1% SDS, 10mM EDTA. Cells were sheared to 300-500 bp fragments (Covaris) and immunoprecipitated with indicated amount of anti-HOPX (sc-398703, SANTA CRUZ) antibody or IgG1 (401401, Biolegend) (1 ug antibody for ChIP-qPCR) overnight. Protein-G magnetic beads (Thermo Scientific) were added to each reaction for 1 hour at 4°C the following day. The magnetic beads were then washed in a series of buffers including a low salt buffer (20 mM Tris-HCl, pH 8, 150 mM NaCl, 2mM EDTA, 0.1% SDS, 1% Triton X-100), a high salt buffer (20 mM Tris-HCl, pH 8.1, 500 mM NaCl, 2 mM EDTA, 0.1% SDS, 1% Triton X-100), a LiCl buffer (10 mM Tris-HCl, pH 8.1, 0.25M LiCl, 1 mM EDTA, 1% NP-40, 1% deoxycholic acid), and finally with TE buffer, with each wash lasting for 5 minutes. Complexes were eluted in 1% SDS, 0.1 M NaHCO3. DNA was decrosslinked and qPCR was performed for regions of interest (Primer sequences are shown in **Supplementary Table 2**).

### Western blot

Proteins were extracted from CD8^+^ T cells (approximately 10^6^) with lysis buffer (50 mM Tris-HCl, pH 7.5, 15 mM EGTA, 100 mM NaCl, 0.1% Triton X-100 and the protease inhibitors). Protein samples were separated on 12% SDS-PAGE and blotted onto PVDF membranes. Blots were incubated with primary antibodies HOPX (sc-398703) and HRP-conjugated secondary antibodies (7076s, CST), and then visualized by the ECL chemiluminescence system (Cytiva).

### T-cell proliferation assay

Sorted T cell subsets were stimulated with anti-CD3 and anti-CD28 antibodies conjugated to microbubbles and placed into either 12-well or 96-well plates at 0.5 × 10^6^ cells/ml for nine days. The transduction with lentivirus (either overexpression or knock-down) was carried out 24 hours post-stimulation. Cells were collected every three days for cell counting and cell proliferation was measured by flow cytometry with Carboxyfluorescein succinimidyl ester (CFSE) dye (Molecular Probes, Eugene, Oregon). Proliferation analysis by CFSE dilution was performed in FlowJo_V10 to calculate replication index. To avoid inter-counter variation, all counting was performed by the same operator.

### Flow cytometry

Sorted T cell subsets were washed once in phosphate-buffered saline (PBS) and diluted to 1 million per 500 μl PBS and 500 μl of PBS with 10 μM CFSE was added to a final cell suspension volume of 1 ml and final concentration of CFSE 5 μM). This was incubated for 10 min at RT, and protected from light, and these labeled T cells were stimulated with anti-CD3/CD28 and analyzed at indicated time. For surface staining, cells were stained with antibodies (CD4, CD8, CD45RA, CD62L from BD Biosciences, dilution 1:200) for 30 min in FACS buffer (HBSS, 0.01% NaN_3_, 0.2%BSA). FACS data collection was done by using a FACScalibur flow cytometer (BD Biosciences). Routinely, 10,000 cells were collected for analysis. Flow cytometry data were analyzed using FlowJo software (Tree Star Inc., Ashland, OR).

### Statistical analysis

Results are shown as the mean ± SEM for at least 3 repeated independent experiments for each group. Student’s t-test was performed to determine statistical differences between 2 groups and two-way analysis of variances (ANOVA) for multiple comparisons using GraphPad. *p* < 0.05 were considered to indicate statistical significance. All results were representative of at least three independent experiments.

## Results

### HOPX is expressed in human naïve T cells and activation increases its expression negatively regulating proliferation

**Figure 1:**
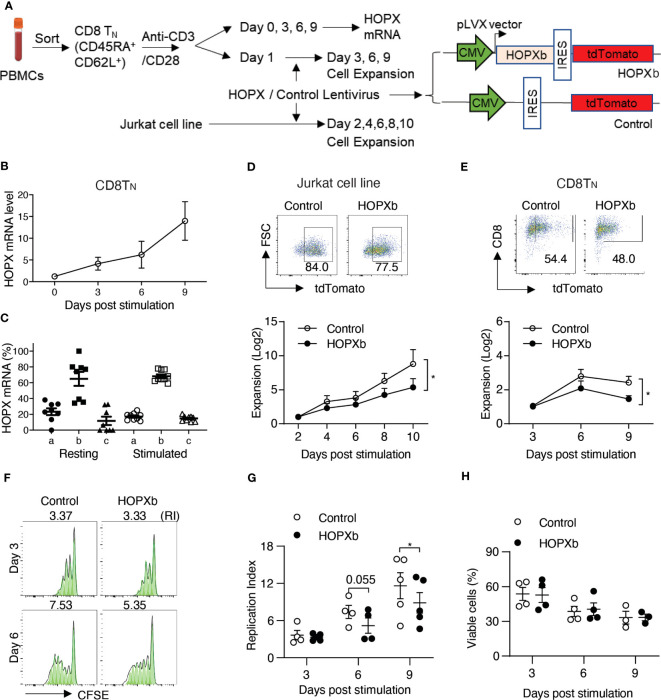
HOPX inhibits the proliferation of naïve human CD8^+^ T cells. **(A)** Experimental scheme. Naïve CD8^+^ T cells (T_N_) (CD62L^+^CD45RA^+^) were isolated from PBMCs of healthy donors using flow cytometry cell sorting and then stimulated *in vitro* by anti-CD3/anti-CD28 antibodies (anti-CD3/CD28). Both freshly isolated and stimulated CD8^+^ T_N_ cells were collected for *HOPX* expression analysis. CD8^+^ T_N_ cells after one day stimulation by anti-CD3/CD28 were used for *HOPX* lentiviral viral transduction for increased HOPX expression analysis. **(B)** *HOPX* expression in CD8^+^ T_N_ cells prior and post anti-CD3/CD28 stimulation *in vitro*. *HOPX* mRNA was determined by quantitative RT-PCR (n=5). *ACOX1* was used as an internal control and the relative change of HOPX mRNA post stimulation was normalized to day 0 (as 1). **(C)** mRNA level of three *HOPX* isoforms (*HOPX-a, b*, and *c*) in freshly isolated and stimulated (two days post-anti-CD3/CD28) CD8^+^ T_N_ cells (n=8). **(D)** Growth of HOPXb overexpressed T cell line. *HOPXb* was cloned into a lentiviral expression vector which carries a TdTomato reporter and the *HOPXb* expressing, and control lentiviruses were used to transduce Jurkat cells. The growth of transduced cells was recorded by cell counts every 2 days during a-ten-day culture and presented as fold change over day 2 for cells expressing tdTomato (n=9). **(E)** Growth of *HOPXb* expressing CD8^+^T_N_ cells. Gating strategy for *HOPXb* expressing and control CD8^+^T_N_ cells based on the expression of tdTomato reporter. CD8^+^T_N_ cells were stimulated with anti-CD3/CD28 at day 0 and transduced with *HOPXb* or control lentivirus on day 1 and then cultured for the nine days. Cell numbers were counted at Day 3, 6 and 9 post stimulation of both *HOPXb* over-expressing and control CD8^+^T_N_ cells based on tdTomato positive signal (n=5). **(F, G)** A representative graph (FlowJo) showed CFSE-labeled T cells at Day 3 and 6 post-anti-CD3/CD28 stimulation in *HOPXb* over-expressing and control CD8^+^T_N_ cells **(F)**. The mean and SEM (n=5) are presented **(G)**. **(H)** Cell viability during 9-day culture. Viability ghost dye was used to stain both *HOPXb* over-expressing and control CD8^+^T_N_ cells at Day 3, 6, and 9 post anti-CD3/CD28 stimulation (n=5). All data in this figure present as mean ± SEM. * as *p*≤ 0.05, ** as *p*≤ 0.01 here and for all other figures.

While repeated T cell activation induces inhibitory receptor expression (5), it is currently unknown if a single round of activation is capable of inducing the negative regulators of T cell activation and proliferation. To investigate this, we analyzed the expression of HOPX during activation of human naïve CD8^+^ and CD4^+^ T cells (T_N_, CD62L^+^CD45RA^+^) isolated from peripheral blood of healthy adults by cell sorting and stimulated with anti-CD3 and anti-CD28 antibodies *in vitro* (**Figure 1A**; **Supplementary Figure 1A**). *HOPX* mRNA was present in freshly isolated T_N_ and increased significantly post-activation (**Figure 1B** and **Supplementary Figure S1B**, *p*=0.021 and 0.036, for CD8^+^ and CD4^+^ T_N_ cells, respectively). It has been reported that human *HOPX* has three major isoforms (11) (**Supplementary Figure 1C**), and we found that all three isoforms were present in different CD8^+^ T_N_ cell subsets with *HOPXb* as the dominant isoform expressed in both freshly isolated (accounting for ~60% of total *HOPX* mRNA) and activated (accounting for ~70% of total *HOPX* mRNA) T cells (**Figure 1C**).

To further investigate the role of HOPX in T_N_ cell activation and proliferation, we used lentiviral transduction to express *HOPX* in a Jurkat T cell reporter cell line, which lacks endogenous *HOPX* expression (**Figure 1A**). After comparing the three isoforms (*HOPXa, b*, and *c*) individually, we found that *HOPXb* exhibited the strongest inhibitory effects on proliferation compared to the control and other isoforms (**Figure 1D**, and **Supplementary Figure 1D**). To investigate if *HOPXb* negatively regulates proliferation in primary T cells, we expressed *HOPXb* via lentiviral transduction in sorted CD8^+^ and CD4^+^ T_N_ cells and stimulated them *in vitro* with anti-CD3/CD28. Again, we found a significant reduction of cell growth in *HOPXb*-transduced T_N_ cells compared to control T_N_ cells for both CD8^+^ and CD4^+^ T_N_ cells (**Figure 1E** and **Supplementary Figure 1E**).

To determine if the reduced cell expansion observed upon overexpression of *HOPXb* was due to decreased proliferation or to increased cell death, we utilized a cell division tracking dye (CFSE) and found a reduction in the replication index (RI) of HOPXb-transduced CD8^+^ T_N_ cells (RI=5.18) compared to control CD8^+^ T_N_ cells (RI=7.39, p=0.055) on Day 6 and RI=8.86 vs 11.61, (p=0.022) on Day 9 (**Figures 1F, G**). Cell viability analysis showed no difference in the percentage of viable cells between *HOPXb*-overexpressing and control CD8^+^ and CD4^+^ T_N_ cells (**Figure 1H**; **Supplementary Figure 1F**). Taken together, we demonstrated that HOPXb negatively regulated activation-induced proliferation of both CD8^+^ and CD4^+^ T_N_ cells by reducing cell cycle progression without affecting their survival.

### HOPXb downregulates key genes involved in T cell proliferation and TCR singling

**Figure 2:**
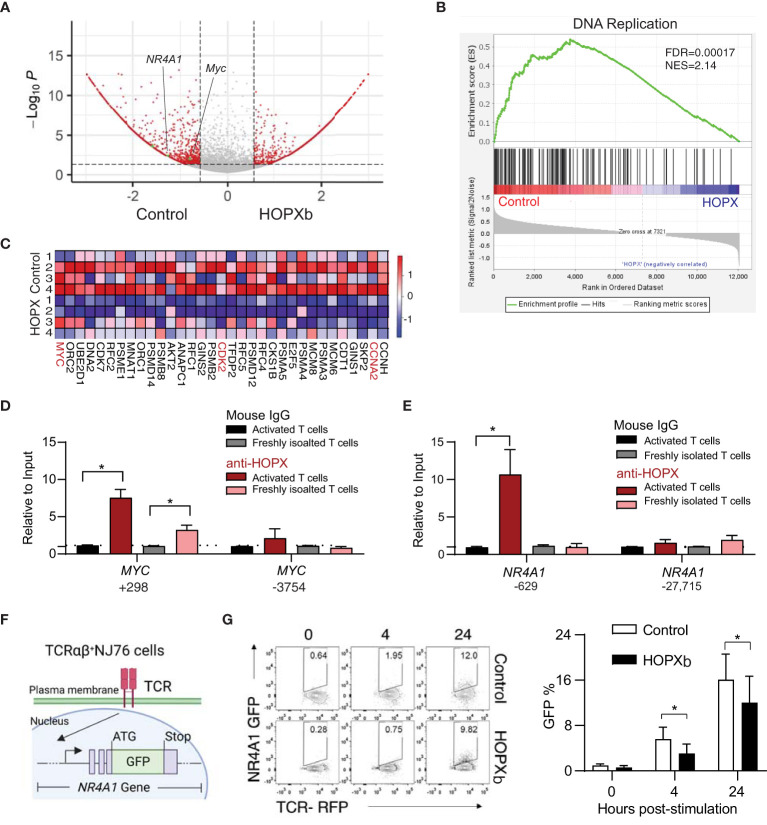
*HOPXb* overexpression induces reduced gene expression related proliferation and TCR signaling in human CD8^+^ T cells. **(A)** *MYC* and *NR4A1* expressions were significantly reduced in HOPXb over-expressing CD8^+^ T cells. Volcano plot based on microarray data shows significantly reduced expressions of *MYC* and *NR4A1*. **(B)** Expression of DNA replication related genes were significantly reduced in *HOPXb* over-expressing compared to the control CD8^+^ T cells using GSEA. The normalized enrichment score (NES) and adjusted p value (FDA) are shown. **(C)** Selected genes whose expressions are significantly changed in *HOPXb* over-expressing compared to the control CD8^+^ T cells. Heat-map of differentially expressed genes derived from GSEA in the DNA replication. Heat scale from high to low (red to blue). **(D)** HOPX binds to the promoter of *MYC* and reduced its expression. qPCR was performed for both positive and negative sites using anti-HOPX and isotype-matching non-specific IgG. **(E)** HOPX binds to the promoter of *NR4A1* and reduced its expression. qPCR was performed for both positive and negative sites using anti-HOPX and isotype-matching non-specific IgG. Data are presented mean ± SEM with p value using Student’s t-test (n=3) for both **(D, E)**. **(F)** Diagram of an αβ TCR NR4A1-GFP reporter cell line (NJ76). NJ76 reporter cells were then transduced with a functional αβ TCR linked to an mCherry reporter via a lentiviral expression vector (TCR-NJ76). **(G)** Over-expression of *HOPXb* reduced NR4A1 mediated TCR signaling. The TCR-NJ76 cell line was transduced with either *HOPXb* or control lentivirus and then were stimulated with anti-CD3/CD28 for 4 and 24 hours. GFP reporter expression was assessed by flow cytometry. Data are presented as mean ± SEM (n=3-4) with p value using Student’s t-test. * as *p*≤ 0.05.

To gain insight into the mechanism underlying *HOPXb*-mediated T_N_ cell proliferation inhibition, we profiled the transcriptome of *HOPXb*-transduced and control CD8^+^ T_N_ cells using microarray analysis. Three days after transduction with *HOPXb*-expressing or control lentivirus, transduced cell expressing a fluorescent reporter (tdTomato^+^) were isolated by cell sorting, and RNA was extracted for microarray analysis (**Supplementary Figure 2A**). We found that 229 genes were upregulated, while 824 genes were downregulated in HOPXb-transduced CD8^+^ T cells (including *MYC*,and *NR4A1*) compared to the control cells (**Figure 2A**). Gene expressions were reduced in *HOPXb* transduced Gene set enrichment analysis revealed that the downregulated genes to be predominantly associated with DNA replication pathway (**Figure 2A**), including key genes such as *MYC*, *CCNA1*, *CCNA2*, *CDC20*, and *CDK2* (**Figures 2B, C**), which was further confirmed by qPCR analysis (**Supplementary Figure 2B**).

HOPX has been shown to interact with serum response factor (SRF) to modulate gene expression (19) and SRF binding sites, also known as CArG boxes, are involved in this process (20). To investigate whether HOPX is directly involved in regulating the expression of *MYC* gene in CD8^+^ T cells, we employed the anti-HOPX chromatin immunoprecipitation (ChIP) method and targeted a site that was indicated to be occupied by SRF (21, 22), located near the transcription start site (TSS) of *MYC* (**Supplementary Figure 2C**). We found a significant enrichment of the predicted DNA fragment from the *MYC* gene in the pull-down of HOPX antibody compared to control (IgG) in both activated (7.3-fold, p=0.03) and freshly isolated CD8^+^ T cells (3.1-fold enrichment) (**Figure 2D**). Taken together, these results support that HOPXb negatively regulates T_N_ cell proliferation by directly repressing MYC expression.

To gain insight into the mechanism underlying *HOPXb*-mediated T_N_ cell proliferation inhibition, we profiled the transcriptome of *HOPXb*-transduced and control CD8^+^ T_N_ cells using microarray analysis. Three days after transduction with *HOPXb*-expressing or control lentivirus, transduced cells expressed a fluorescent reporter (tdTomato^+^) were sorted, and RNA was extracted for microarray analysis. We found that 229 genes were upregulated, while 824 genes were downregulated in HOPXb-transduced cells compared to the control cells (**Supplementary Figure 2A**). Gene set enrichment analysis revealed that the downregulated genes to be predominantly associated with DNA replication pathway (**Figure 2A**), including key genes such as *MYC*, *CCNA1*, *CCNA2*, *CDC20*, and *CDK2* (**Figures 2B, C**), which was further confirmed by qPCR analysis (**Supplementary Figure 2B**).

HOPX has been shown to interact with serum response factor (SRF) to modulate gene expression (19) and SRF binding sites, also known as CArG boxes, are involved in this process (20). To investigate whether HOPX is directly involved in regulating the expression of *MYC* gene in CD8^+^ T cells, we employed the anti-HOPX chromatin immunoprecipitation (ChIP) method and targeted a site that was indicated to be occupied by SRF (21, 22), located near the transcription start site (TSS) of *MYC* (**Figure 2E**). We found a significant enrichment of the predicted DNA fragment from the *MYC* gene in the pull-down of HOPX antibody compared to control (IgG) in both activated (7.3-fold, p=0.03) and freshly isolated CD8^+^ T cells (3.1-fold enrichment) (**Figure 2F**). Taken together, these results support that HOPXb negatively regulates T_N_ cell proliferation by directly repressing MYC expression.

### HOPXb reduces TCR activation signaling via directly repressing NR4A1 expression

The proliferation of T cells induced by TCR activation is a tightly regulated process that requires the activation and re-localization of multiple transcription factors (23). *NR4A1* is an immediate-early gene following T cell receptor ligation (24) and a key regulator for T cell functions (25, 26). We found a significant reduction of *NR4A1* expression in the *HOPXb*-transduced CD8^+^ T cells compared to the control (**Figure 2A** and **Supplementary Figure 2D**) and identified a distinct site within the promoter region of *NR4A1* from analyzing the published SRF CHIP-seq dataset (22) (**Supplementary Figure 2E**). To confirm whether HOPX/SRF directly binds to the NR4A1 promoter, we performed ChIP-qPCR using anti-HOPX antibody and showed a significant enrichment (9.6-fold, *p*=0.05) of the DNA fragment containing the reported HOPX/SRF-binding site in the *NR4A1* promoter region in activated CD8^+^ T cells (**Figure 2E**), providing evidence that HOPX/SRF directly regulates *NR4A1* expression in CD8^+^ T cells. To further determine the regulatory role of HOPXb on *NR4A1* during TCR activation, we generated a TCR-signaling reporter system (NJ76-TRAV8-6-TRAJ42) by introducing a functional TCR in tandem with an mCherry reporter into NR4A1-GFP (NJ76) cell line (27) (**Figure 2F**). The *HOPXb*-tdTomato^+^ and control constructs were delivered into these cells by viral transduction. We compared the strength of GFP signal between *HOPXb* and control transduced NJ76-TRAV8-6-TRAJ42 cells after anti-CD3/CD28 stimulation and found a significant reduction of GFP level in *HOPXb*-transduced cells compared to the control cells (**Figure 2G**). This suggests that HOPXb represses both cell cycle-related genes (*MYC*) and an immediate-early TCR activation-induced gene and TCR signaling (*NR4A1*).

### HOPX regulates CD8^+^ T cell differentiation-associated reduced proliferation

**Figure 3:**
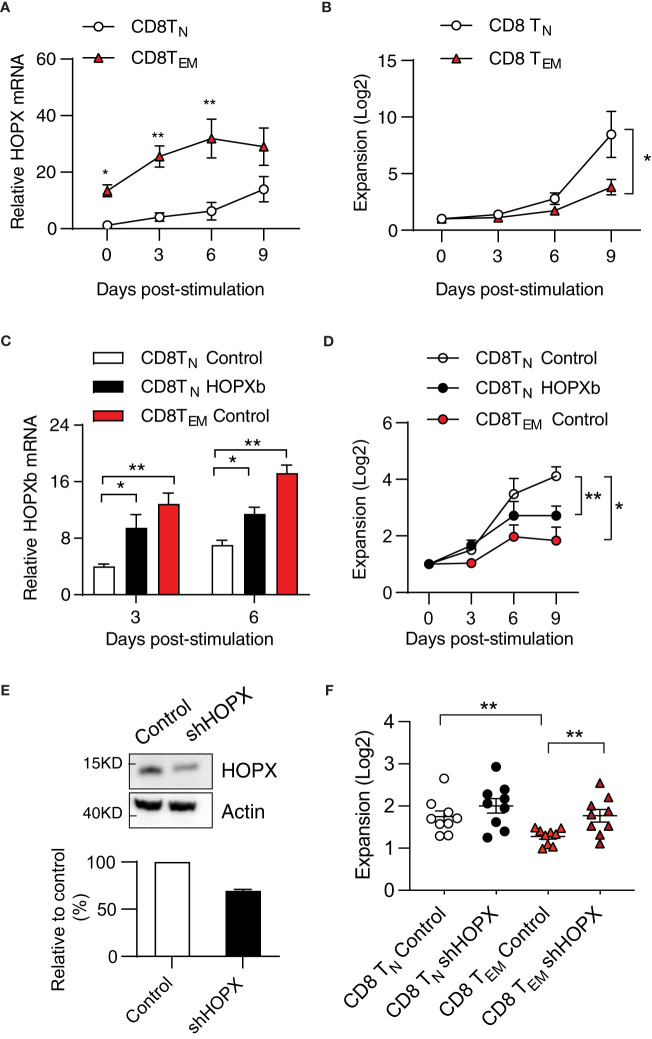
HOPX contributes to the proliferation difference from human CD8^+^T_N_ to T_EM_ cells in response to stimulation. **(A)** Higher *HOPX* mRNA level in CD8^+^ T_EM_ than in CD8^+^ T_N_ cells before and after *in vitro* stimulation. CD8^+^ T_N_ (CD62L^+^CD45RA^+^) and T_EM_ (CD62L^-^CD45RA^-^) cells were isolated from healthy donors by cell sort and stimulated (anti-CD3/CD28) and harvested at day 0, 3, 6, and 9. The expression of *HOPX* mRNA in different T cell subsets was measured by qRT-PCR and normalized to the level of *ACOX1*. The mean ± SEM are present (n=5). Two-way ANOVA was used to calculate p value between two conditions. **(B)** Growth of CD28^+^ CD8^+^ T_N_ and CD28^+^ CD8^+^ T_EM_ cells *in vitro* post stimulation (anti-CD3/CD28). Stimulated cells were counted at the indicated days and the mean ± SEM are present (n=7). **(C)** *HOPX* mRNA level in HOPXb and control transduced CD8^+^ T_N,_ and control transduced T_EM_ cells *in vitro*. TdTomato^+^ cells were isolated by cell sorter from *HOPX* over-expressing or control lentiviral transduced CD8^+^ T_N,_ and T_EM_ cells. *HOPX* mRNA level was measured in control CD8^+^T_N_, CD8^+^T_EM_, and *HOPXb* over-expressing CD8^+^T_N_ cells by qRT-PCR at day 3 and 6 post stimulation and normalized to *ACOX1*. The mean ± SEM are present (n=3-4). **(D)** Growth of *HOPXb* over-expressing CD8^+^T_N_ cells and control CD8^+^T_N_ and T_EM_ cells *in vitro*. Cell numbers were counted based on tdTomato positive cells at day 3, 6, and 9. The mean ± SEM are present (n=5). Two-way ANOVA was used to calculate p value between two conditions. **(E)** Reduced HOPX protein by shRNA knockdown. A shRNA (target on exon2 of *HOPX*) was designed and cloned into a lentiviral vector and then transduced Jurkat cell line and the efficiency of shRNA-mediated knockdown was confirmed by Western blot. A representative graph (Western blot) showing the level of HOPX in HOPX-transduced Jurkat cells 3 days after treatment with either control or HOPX shRNA and quantification (20-30% reduction). **(F)** Increased growth of CD8^+^T_EM_ cells post HOPX knockdown. CD8^+^T_EM_ and CD8^+^T_N_ cells were isolated, stimulated, and transduced with HOPX shRNA and control lentiviruses. Cell numbers were counted at day 2 post viral transduction and the mean ± SEM are present (n=9) with p value using Student’s t-test. * as *p*≤ 0.05, ** as *p*≤ 0.01.

It is reported that *HOPX* expression increases from T_N_ to memory (T_EM_) T cells (13), but its role in naïve and memory T cells has not been fully characterized. We hypothesize that HOPX regulates the proliferation behavior of different T cell subsets. To directly test this, we analyzed the proliferation of CD8^+^ T_N_ (CD28^+^/CD45RA^+^/CD62L^+^) and T_EM_ (CD28^+^/CD45RA^-^/CD62L^-^) cells following anti-CD3/CD28 antibody stimulation *in vitro* and found that *HOPX* mRNA levels were four-five times higher in T_EM_ cells than in T_N_ cells (0 - 6 days post-stimulation (**Figure 3A**). Correspondingly, CD8^+^ T_EM_ cells exhibited a significantly slower growth than CD8^+^ T_N_ cells over 9-day culture (**Figure 3B**).

To investigate whether the elevated expression of *HOPX* causes reduced the proliferative capacity of CD8^+^ T_EM_ cells, we first compared expression of *HOPXb* in transduced and control T_N_ cells and control T_EM_ cells. In comparison to the control-virus-transduced CD8^+^ T_N_ cells, we observed approximately a 2-fold increase in HOPXb expression in HOPXb-transduced CD8^+^ T_N_ cells and a 3-fold increase in activated CD8^+^ T_EM_ cells post 3- and 6-day stimulations (**Figure 3C**). We found that *HOPXb* level correlates well with activation-induced proliferation of control T_N_ cells, *HOPXb*-transduced T_N_ cells, and T_EM_ cells (**Figure 3D**). We then reduced expression of *HOPX* in T cells via shRNA targeting exon 2 of *HOPX* (**Supplementary Figure 3**). To verify the efficiency of HOPX knockdown in T cells, we transduced the knockdown vector into HOPX-expressing Jurkat T cells and observed a 20-30% reduction in the protein level of HOPX 3 days post-transduction (**Figure 3E**). When we used shRNA to knockdown *HOPX* expression in CD8^+^ T_EM_, we observed a 1.4-fold increase in cell number in *shHOPX*-transduced CD8^+^ T_EM_ cells compared to control cells (**Figure 3F**). Importantly, knockdown of *HOPX* eliminated the reduced proliferation of CD8^+^ T_EM_ cells compared to that of CD8^+^ T_N_ cells (**Figure 3F**). Collectively, these results show that the level of *HOPXb* determines the degree of activation induced proliferation of CD8^+^ T_N_ and T_EM_ cells.

### Elevation of *HOPX* expression explains reduced activation-induced proliferation of CD8^+^ T cells in old adults

**Figure 4:**
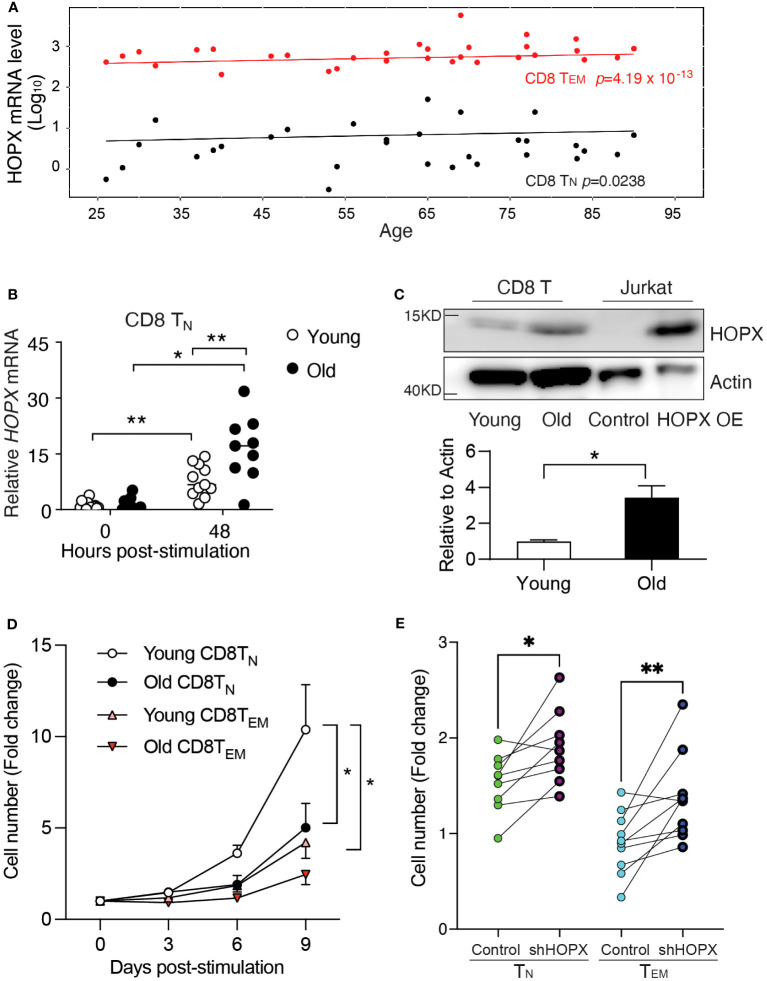
Increased *HOPX* in resting and activated human CD8^+^ T cell subsets in older adults causes reduced activation-induced proliferation. **(A)** Increased expression of *HOPX* in human CD8^+^ T_N_ and T_EM_ cells with age. scRNAseq data were from the published data (31). In brief, T_N_ (22,694 cells from 30 samples) and T_EM_ (27709 cells from 30 samples) cells expressed *HOPX* and its expression values were used to create separate mixed-linear effect regression models for each donor using the lme4 package, with *HOPX* as the dependent variable, Age and Sex as independent variables, and a random effect term (formula: HOPX ~ Age + Sex + (1|random_effect)). The mean *HOPX* expression for each sample was plotted against age, and the trend line is modeled by mixed effects linear regression with p values for T_N_ and T_EM_ cells. **(B)** Increased *HOPX* mRNA levels in CD8^+^ T_N_ cells post stimulation (anti-CD3/CD28) with age. *HOPX* mRNA levels were measured by qRT-PCR in CD8^+^ T_N_ cells both from young (<40 years old) and old (>70 years old) donors with rest or anti-CD3/CD28 activation for 48 hours. **(C)** Increased HOPX protein level in CD8^+^ T cells in older adults. Human CD8^+^ T cells were collected 48 hours post-stimulation from young and old subjects and cell lysate were used for Western blot with anti-HOPX and anti-Actin antibodies as the loading control. The HOPXb over-expressing Jurkat T cell line was used as a positive control. A representative gel image (upper), and data quantified with ImageJ (lower) (n=4). **(D)** Reduced expansion of CD28^+^ CD8^+^ T_N_ and CD8^+^ T_EM_ cells in older adults post-stimulation with anti-CD3 and CD28. CD8^+^ T_N_ and T_EM_ cells were isolated from healthy young and old donors and stimulated with anti-CD3/CD28 and cell numbers were counted at day 3, 6, and 9. The mean ± SEM are present (n=5-8). Two-way ANOVA was used to calculate p value between two conditions. **(E)** Knockdown of *HOPX* via lentiviral transduction of shRNA increased proliferation of CD28^+^CD8^+^ T_EM_ cells and CD28^+^CD8^+^ T_N_ cells 48h post-transduction (n=9 for T_N_ and n=10 for T_EM_; ≥70 years old). Data are mean ± SEM with p value using paired Student’s t-test. * as *p*≤ 0.05, ** as *p*≤ 0.01.

Age-related changes in CD8^+^ T cell responses are, in part, caused by the reduced activation-induced proliferation of CD8^+^ T_N_ cells (28–30). Our recent study has identified over 1,500 genes whose expressions significantly increased in human CD8^+^ T cell subsets over 70 years of adult life. Many of these genes are involved in both immune functions (antigen processing and presentation, exocytosis, etc.) and general cellular functions (oxidant detoxification, oxidative phosphorylation, etc.) (31). Interestingly, we observed a significant increase in HOPX mRNA levels with age in both CD8^+^ T_N_ cells (22,603 cells from 22 adults, p=0.024) and T_EM_ cells (27,709 cells from 22 adults, p<0.001) in this published dataset (31) (**Figure 4A**). To determine if such age-related changes remain post-activation, we isolated and stimulated (anti-CD3/CD28) CD8^+^ T cell subsets from young (age<40 years old, n=8) and old donors (age≥70 years old, n=8) (**Supplementary Figure 4A**). In stimulated CD8^+^ T_N_ cells (**Figure 4B**), we found significantly higher levels of *HOPX* mRNA in old than in young donors, whiles this was not the case in stimulated memory CD8^+^ subsets (T_CM_ and T_EM_ cells) (**Supplementary Figures 4B, C**). Again, *HOPXb* was the most abundant HOPX isoform in CD8^+^ T cell subsets of both young and old donors (**Supplementary Figures 4D–F**). We further confirmed the age-associated increase in mRNA level resulting in increased protein in activated CD8^+^ T_N_ cells from old compared to those from young donors (**Figure 4C**). Functionally, we found that CD28^+^CD8^+^ T_N_ cells from old donors were significantly less expanded after *in vitro* stimulation than those from young donors (**Figure 4D**). To further determine if activation-induced proliferation of T cells from old adults could be improved by reduced HOPX expression, we applied the shRNA mediated *HOPX* knockdown in CD8^+^ T_N_ and T_EM_ cells isolated from healthy old adults (Age≥70 years old). shRNA and control virus transduction yielded comparable percentage of reporter (EGFP^+^) (**Supplementary Figure 4G**). We found that reduced HOPX expression resulted in improved proliferation in both CD8^+^ T_N_ (1.2-fold increase, p=0.013, n=9) and CD8^+^ T_EM_ (1.5-fold increase, p=0.009, n=10) cells compared to controls (**Figure 4E**). The proliferations of shRNA treated CD8^+^ T_N_ and T_EM_ from old adults were comparable to the control treated CD8^+^ T_N_ and T_EM_ from young adults. Taken together, increased HOPX expression with age contributed to the reduced activation-induced expansion of CD8^+^ T_N_ and T_EM_ cells.

## Discussion

Signaling pathways following the engagement of TCR and co-stimulatory receptors have been extensively studied, elucidating the collective impact of inducing T cells from a resting state to proliferation and differentiation, thereby fulfilling their function. While positive signals are well-documented, our understanding of negative signals and their regulators during T cell activation is limited. In this study, we unveil HOPX as a crucial negative regulator in human T cell activation. We observe the expression of three *HOPX* isoforms, with *HOPXb* being the most abundant in both resting and activated T cells. The levels of *HOPX* mRNA increase from T_N_ to T_CM_ to T_EM_, corresponding to the incrementally reduced robustness of activation-induced proliferation. Moreover, manipulating HOPX levels in human primary CD8^+^ T_N_ and T_EM_ cells, either by increasing (lentiviral mediated expression) or reducing (shRNA), results in decreased or increased proliferation, respectively. Mechanistically, we demonstrate that HOPX directly binds to the promoters of two critical genes (*MYC* and *NR4A1*) in T cell proliferation and signaling, suppressing their expressions. Additionally, our findings reveal that aging is associated with a significant increase in HOPX expression in CD8^+^ T_N_ and T_EM_ cells. Notably, reducing HOPX levels leads to a substantial increase in the proliferation of these T cells. In summary, our study identifies HOPX as a pivotal negative regulator of T cell activation, shedding light on its role in T cell differentiation and aging.

While the specific partner of HOPX in human CD8^+^ T cells remains unconfirmed, the overexpression of *HOPXb* induces alterations in the expression of over 1000 genes, with notable impacts on crucial genes such as *MYC* and *NR4A1*. Our findings demonstrate that HOPX binds to the promoters of *MYC* and *NR4A1*, resulting in a reduction of their expression in activated CD8^+^ T cells. This downregulation of *MYC* and *NR4A1* in activated CD8^+^ T cells by HOPX significantly diminishes proliferation and weakens TCR signaling, delivering a repressive signal post-TCR-mediated activation. However, the impact of other changed genes in T cell activation and proliferation remains to be further determined.

Therefore, identification of the partner of HOPX in T cells and further exploration of genes regulated by HOPX will provide a better understanding of the negative signaling pathway and its transcriptome regulation in T cell activation. Moreover, the regulatory mechanisms governing HOPX expression in T cells during differentiation and its dysregulation during aging remain unknown. Unraveling these aspects is crucial for a more profound comprehension of the molecular mechanisms underlying both activation and inhibition processes in T cell activation, particularly how these processes evolve during aging. A comprehensive understanding of the regulation and function of HOPX is essential for elucidating the intricate dynamics of T cell activation and the impact of aging on these processes.

## Data availability statement

The datasets presented in this study can be found in online repositories. The names of the repository/repositories and accession number(s) can be found here: GSE234697 (GEO).

## Ethics statement

The studies involving humans were approved by National Institutes of Health Institutional Review Boards. The studies were conducted in accordance with the local legislation and institutional requirements. The participants provided their written informed consent to participate in this study.

## Author contributions

QY: Conceptualization, Data curation, Formal Analysis, Methodology, Writing – original draft, Writing – review & editing, Investigation, Validation, Visualization. MP: Methodology, Writing – review & editing, Data curation, Investigation. JL: Investigation, Methodology, Writing – review & editing. JC: Formal Analysis, Writing – review & editing. YZ: Data curation, Formal Analysis, Writing – review & editing. HH: Data curation, Formal Analysis, Writing – review & editing. EL: Data curation, Methodology, Writing – review & editing. SD: Data curation, Supervision, Writing – review & editing. N-pW: Formal Analysis, Methodology, Supervision, Writing – original draft, Writing – review & editing, Conceptualization, Funding acquisition, Resources, Visualization.
