# Supplementary material for: Homeodomain-only protein suppresses proliferation and contributes to differentiation- and age-related reduced CD8+ T cell expansion
Source: Front Immunol. 2024 Feb 12;15:1360229. doi: 10.3389/fimmu.2024.1360229 (PMC10895957; doi:10.3389/fimmu.2024.1360229)
Supplement: Supplementary file 1 [file DataSheet_1.docx]

**Homeodomain-only protein (HOPX) suppresses proliferation and contributes to differentiation- and age-related reduced CD8^+^ T cell expansion**

**Running title**

HOPX negatively regulates human CD8^+^ T cells proliferation

Qian Yang^1^, Michael Patrick^1^, Jian Lu^1^, Joseph Chen^1^, Yongqing Zhang^2^, Humza Hemani^1^, Elin Lehrmann^2^, Supriyo De^2^, and Nan-ping Weng^1*^

^1^Laboratory of Molecular Biology and Immunology, National Institute on Aging, National Institutes of Health, USA

^2^Laboratory of Genetics and Genomics, National Institute on Aging, National Institutes of Health, USA

Supplementary Fig.1 Analysis of HOPX expression in human CD8^+^ and CD4^+^ T cells.

Supplementary Fig.2 Experimental design of *HOPXb* overexpression induces reduced gene expressions in human CD8^+^ T cells.

Supplementary Fig.3 Construct and sequence of HOPX knockdown by shRNA.

Supplementary Fig.4 HOPX expression in CD8^+^ T cell subsets and in young and old adults.

Supplementary Table 1. Donor information used in Figures.

Supplementary Table 2. List of primer pairs used for qRT-PCR and CHIP-qPCR.


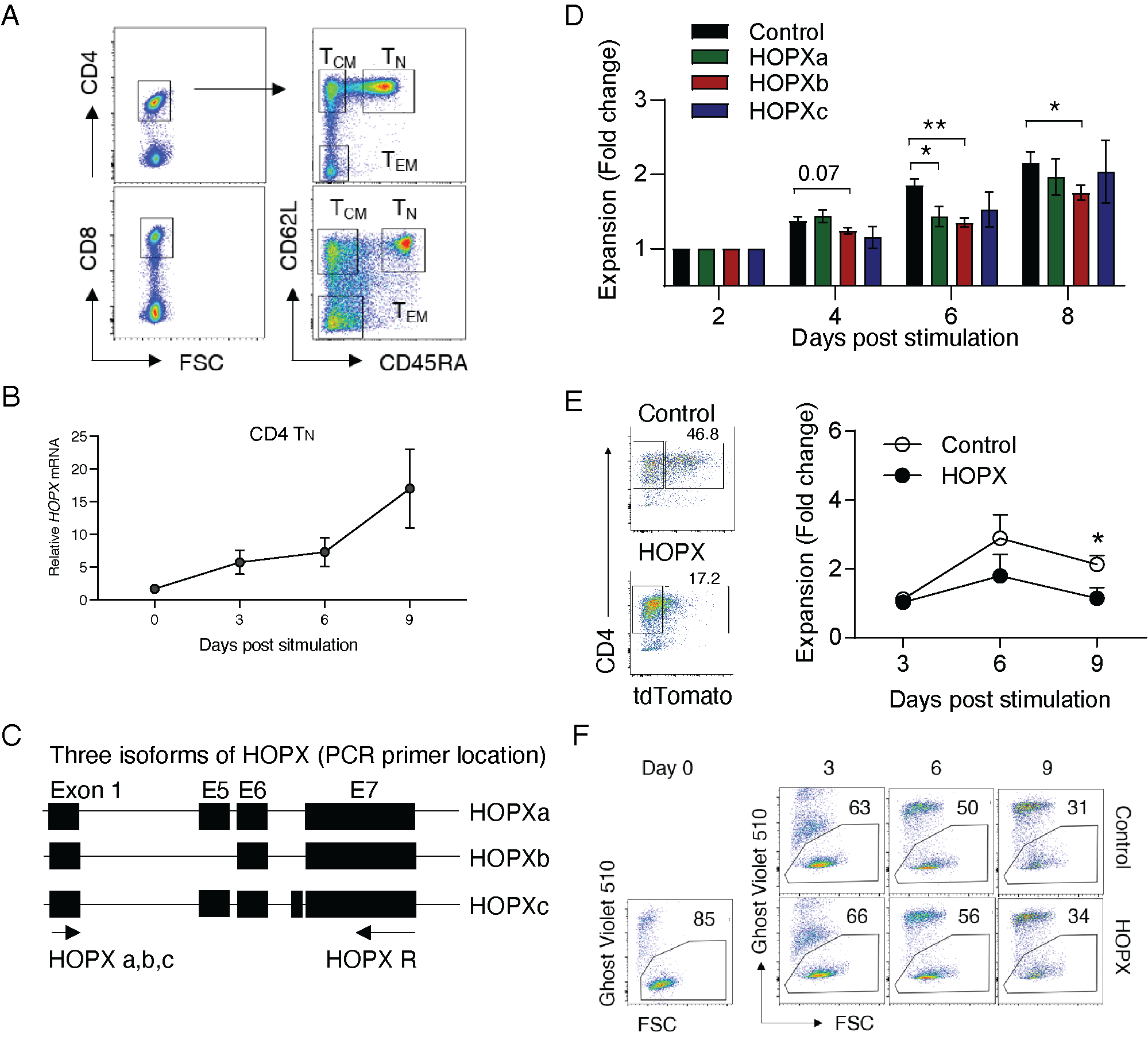


**Supplementary Fig.1 Analysis of HOPX expression in human CD8^+^ and CD4^+^ T cells.**

(A) Gating strategy for isolating CD8^+^ and CD4^+^ T cell subsets subtype from PBMCs of healthy donors were sorted based on the surface marker (CD62L and CD45RA). (B) Expression of HOPX in CD4^+^T cell different subsets treated with anti-CD3/CD28 antibodies *in vitro* and data collected at indicated days. *ACOX1* was used as an internal control. n=5 in each group. (C) Scheme of three isoforms of HOPX mRNA and location of RT-PCR primers are marked by arrow line. (D) Consistent cell culture *in vitro*, cell count was performed every 2 days in HOPXa, b, c OE and control group of the Jurkat T cells. The cell number fold change was calculated based on tdTomato positive cell number of day 2 (n=9). (E) Gate strategy of HOPX overexpression and control in CD4^+^ T cells (left) and cell expansion *in vitro* of HOPX overexpression and control in CD4^+^ T cells (right). (F) Cell viability was assessed by Ghost violet 510 dye using flow cytometer. Percentages of viable cells are shown. *p*-values are calculated as t-test where *as *p*≤ 0.05, **as *p*≤ 0.01.

**Supplementary Fig.2 Experimental design of *HOPXb* overexpression induces reduced gene expressions in human CD8^+^ T cells** (A) Experimental design of transcriptome analysis of *HOPXb* over-expressing human CD8^+^ T_N_ cells. CD8^+^T_N_ cells were harvested from healthy donors (n=4), stimulated with anti-CD3/CD28, and transduced by *HOPXb* over-expressing and control lentivirus at day 1 post stimulation. TdTomato positive cells were isolated by the cell sorter and mRNA were further isolated for microarray analysis (n = 4). Data were normalized with the quantile method and filtered for probes detected in at least two-third of replicates for each condition. (B) Confirmation of altered cell cycle regulated gene expression (*MYC/CCNA1/CCNA2/CDC20/CDK2)* in *HOPXb* over-expressing CD8^+^ T cells by quantitative RT-PCR. (C) Experimental scheme of ChIP-qPCR. Freshly isolated and stimulated (anti-CD3/CD28) CD8^+^ T_N_ cells were generated for anti-HOPX ChIP assay. Two known sites: positive promoter site (-298 bp) and negative remote site (-3754) were selected for evaluating the interaction between HOPX and *MYC.* (D) Confirmation of reduced *NR4A1* expression in CD8^+^ T cells by qRT-PCR. *HOPXb* over-expressing CD8^+^ T cells were sorted by tdTomato and CD8 positive cells after 2 days post transduction with *HOPXb* and control viruses and RNA were extracted from sorted cells for qRT-PCR analysis (n=7). (E) Scheme of the positive (-629 bp from TSS) and negative (-27,715 bp) sites of *NR4A1* gene used by ChIP-qPCR.

**
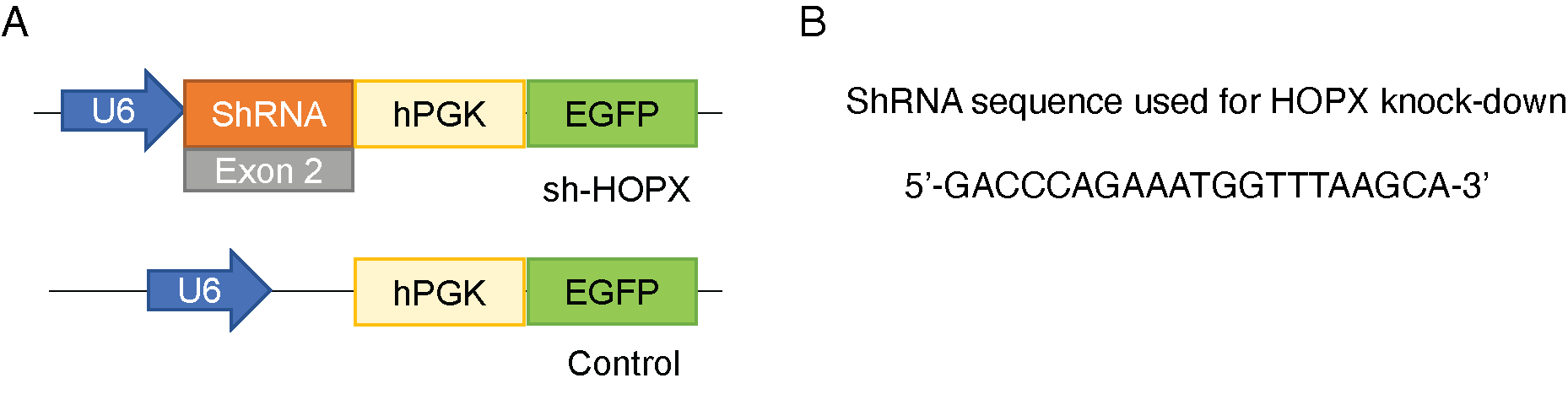
 Supplementary Fig.3 Construct and sequence of HOPX knockdown by shRNA.**

(A) Construct of ShRNA of HOPX in a lentiviral vector with EGFP reporter. (B) Oligo sequence of HOPX used for knockdown.


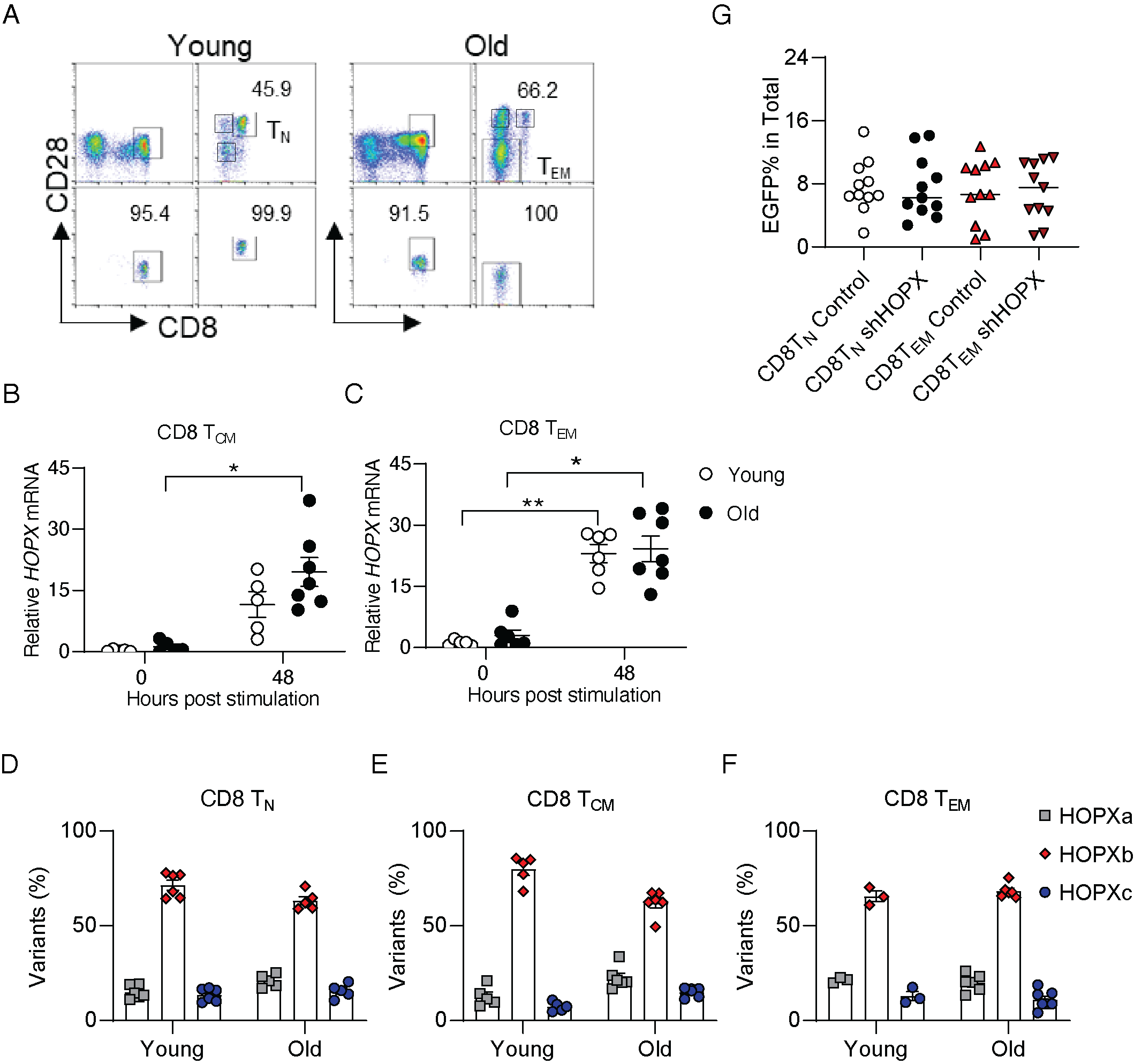


**Supplementary Fig.4 HOPX expression in CD8^+^ T cell subsets and in young and old adults.**

(A) Gating strategy of CD8^+^/CD28^+^ T_N_, T_CM_ and T_EM_ cells from young and old adults. (B) HOPX expression in CD8^+^ T_CM_ and (C) T_EM_ cells before and 48 hours post anti-CD3/CD28 stimulation. *HOPX* mRNA was determined by quantitative RT-PCR. *ACOX1* was used as an internal control for normalization. (D) HOPX isoforms expression in CD8^+^ T_N_, (E) T_CM_ and (F) T_EM_ cells from young (n=3-6) and old (n=5-6) adults. *HOPX* isoform mRNA was determined by quantitative RT-PCR and their size was resolved by agarose gel electrophoresis (see S. Fig1C). (G) EGFP reporter expressions in HOPX or control knockdown vector transduced CD8 T_N_ and T_EM_ cells (n=11) (Day 3 post anti-CD3/CD28 stimulation) were determined by flow cytometry. Percentages of EGFP+ cells were presented.

| **Supplementary Table 1 Donor information used in Figures** | | | |
| --- | --- | --- | --- |
| Donor | Age | Sex | Used in Figure (s) |
| H001 | 44 | F | Fig 1B, Fig 4A |
| H002 | 45 | M | Fig 1B, Fig 4A |
| H003 | 49 | F | Fig 1B, Fig 4A |
| H004 | 55 | M | Fig 1B, Fig 4A |
| H005 | 29 | F | Fig 1B, Fig 4A, Fig 5B |
| H006 | 31 | M | Fig 2A |
| H007 | 42 | M | Fig 2A |
| H008 | 43 | F | Fig 2A |
| H009 | 56 | F | Fig 2A |
| H010 | 58 | F | Fig 2A |
| H011 | 32 | F | Fig 2F |
| H012 | 41 | F | Fig 2F |
| H013 | 42 | F | Fig 2F |
| H014 | 54 | M | Fig 2F |
| H015 | 44 | F | Fig 2F, Fig, 4F |
| H016 | 32 | F | Fig 3B |
| H017 | 41 | F | Fig 3B |
| H018 | 42 | M | Fig 3B |
| H019 | 43 | F | Fig 3B |
| H020 | 56 | F | Fig 3B |
| H021 | 58 | F | Fig 3B |
| H022 | 31 | M | Fig 4B |
| H023 | 33 | F | Fig 4B |
| H024 | 34 | F | Fig 4B |
| H025 | 35 | M | Fig 4B |
| H026 | 41 | M | Fig 4B |
| H027 | 69 | M | Fig 4B |
| H028 | 26 | F | Fig 4B, D, F |
| H029 | 74 | F | Fig 4B, Fig 5D |
| H030 | 34 | F | Fig 4F |
| H031 | 41 | M | Fig 4F |
| H032 | 44 | M | Fig 4F |
| H033 | 56 | F | Fig 4F |
| H034 | 58 | M | Fig 4F |
| H035 | 59 | F | Fig 4F |
| H036 | 79 | M | Fig 4F |
| H037 | 27 | F | Fig 5B |
| H038 | 28 | F | Fig 5B |
| H039 | 31 | F | Fig 5B |
| H040 | 31 | M | Fig 5B |
| H041 | 32 | M | Fig 5B |
| H042 | 33 | F | Fig 5B |
| H043 | 33 | M | Fig 5B |
| H044 | 34 | M | Fig 5B |
| H045 | 35 | M | Fig 5B |
| H046 | 47 | M | Fig 5B |
| H047 | 48 | M | Fig 5B |
| H048 | 68 | M | Fig 5B |
| H049 | 72 | F | Fig 5B |
| H050 | 77 | M | Fig 5B |
| H051 | 80 | M | Fig 5B |
| H052 | 81 | M | Fig 5B |
| H053 | 82 | M | Fig 5B |
| H054 | 70 | M | Fig 5B, C |
| H055 | 72 | M | Fig 5B, C, D, E |
| H056 | 83 | F | Fig 5B, C, D, E |
| H057 | 75 | F | Fig 5B, C, E |
| H058 | 22 | M | Fig 5C |
| H059 | 23 | F | Fig 5C |
| H060 | 23 | M | Fig 5C |
| H061 | 25 | M | Fig 5C |
| H062 | 26 | F | Fig 5D |
| H063 | 31 | M | Fig 5D |
| H064 | 33 | F | Fig 5D |
| H065 | 34 | F | Fig 5D |
| H066 | 35 | M | Fig 5D |
| H067 | 41 | M | Fig 5D |
| H068 | 70 | F | Fig 5D |
| H069 | 79 | F | Fig 5D |
| H070 | 71 | F | Fig 5D, 5E |
| H071 | 79 | M | Fig 5D, E |
| H072 | 86 | M | Fig 5D, E |
| H073 | 70 | F | Fig 5E |
| H074 | 77 | F | Fig 5E |

**Supplementary Table 2 Primer sequences**

| qPCR primer |  |
| --- | --- |
| HOPX-F | GACAAGCACCCGGATTCCA |
| HOPX-R | GTCTGTGACGGATCTGCACTC |
| HOPX isoform F | CAAACCCAGGGCTTGCGCTT |
| HOPX isoform R | GCGGAGGAGAGAAACAGAGAT |
| MYC-F | CTCCCTCCACTCGGAAGGA |
| MYC-R | CGGTTGTTGCTGATCTGTCTCA |
| CCNA1-F | CAGCTTTTTGCCTGGCAAACT |
| CCNA1-R | TCACTCAGGCAAGGCACAATTT |
| CCNA2-F | ACAAAGCTGGCCTGAATCATTAAT |
| CCNA2-R | AGGTAGGTCTGGTGAAGGTCCAT |
| CDC20-F | GCACAGTTCGCGTTCGAGA |
| CDC20-R | CTGGATTTGCCAGGAGTTCGG |
| CDK2-F | TGCTCTCACTGGCATTCCTCTT |
| CDK2-R | TTTAAGGTCTCGGTGGAGGACC |
| NR4A1-F | GCAAGTGGGCGGAGAAGAT |
| NR4A1-R | TCGCCTGGCTTAGACCTGTAC |
| ACOX1-F | TGCTTTGGTTGATGCATTTGA |
| ACOX1-R | CATAGCGGCCAAGCACAGA |
| CHIP-qPCR primer |  |
| MYC(-3754)-F | TTTTAGAGATGGCACGTCACC |
| MYC(-3754)-R | ACCAAAGTGATACGTGTCCA |
| MYC (+298)-F | CCTTGCCGCATCCACGAAA |
| MYC (+298)-R | CCAAATGGGCAGAATAGCCTC |
| NR4A1 (-27715)-F | GGTGGCCGTCTCCCTACAGA |
| NR4A1 (-27715)-R | CGATACCCAGCCAGACGACA |
| NR4A1 (-629)-F | TTTCCCAACTAGGGTGCACT |
| NR4A1 (-629)-R | GTTAGAGCCCTCGCTTAGTCC |
